# Effect of nutrition education integrating the health belief model and theory of planned behavior on dietary diversity of pregnant women in Southeast Ethiopia: a cluster randomized controlled trial

**DOI:** 10.1186/s12937-023-00907-z

**Published:** 2024-01-03

**Authors:** Girma Beressa, Susan J Whiting, Tefera Belachew

**Affiliations:** 1 Department of Public Health, School of Health Sciences, Madda Walabu University, Goba, Ethiopia; 2 Nutrition and Dietetics Department, Faculty of Public Health, Jimma University, Jimma, Ethiopia; 3 College of Pharmacy and Nutrition, University of Saskatchewan, Saskatoon, Canada

**Keywords:** Dietary diversity, Ethiopia, GEE, Nutrition education, Pregnant women

## Abstract

**Background:**

Maternal anemia, miscarriage, low birth weight (LBW), preterm birth (PTB), intrauterine growth restriction (IUGR), prenatal and infant mortality, morbidity, and the risk of chronic disease later in life are all increased by a lack of dietary diversity during pregnancy. However, evidence for the effect of nutrition education on the dietary diversity score (DDS) among pregnant women was sparse in Ethiopia, particularly in the study areas. This study aimed to assess the effect of nutrition education on dietary diversity among pregnant women in urban settings in Southeast Ethiopia.

**Methods:**

A community-based two-arm parallel cluster randomized controlled trial was conducted among 447 randomly selected pregnant women attending antenatal care (224 intervention group and 223 control group) at health facilities from February to December 2021. A multistage cluster sampling technique, followed by systematic sampling, was used to select the pregnant women. Pregnant women who participated in the interventions were given nutrition education starting at 16 weeks of gestation and continuing for 6 months. We used a pre-tested, interviewer-administered, structured questionnaire to collect the data. A 24-hour qualitative dietary recall was used to calculate the dietary diversity score (DDS). A multivariable generalized estimating equation (GEE) model was conducted to evaluate the intervention effect.

**Results:**

After the intervention, the proportion of adequate dietary diversity was 14.15% higher in the intervention arm compared to the control group (45.09% versus 30.94%, *P* = 0.002). The overall difference in adequate dietary diversity between the two groups was 8.5%. After adjusting for background characteristics, the multivariable GEE binary logistic model revealed that having received intervention [(AOR = 1.89, 95% CI: 1.27, 2.79)], being literate [(AOR = 3.41, 95% CI: 1.13, 10.23)], and having high wealth [(AOR = 1.60, 95% CI: 1.09, 2.35)] significantly improved adequate dietary diversity.

**Conclusion:**

The findings indicated that having received the intervention, being literate, and having a high level of wealth significantly improved maternal dietary diversity. Efforts should be made to increase nutrition education using the health belief model (HBM) and the theory of planned behavior (TPB). Moreover, there is a need to improve literacy and economic empowerment through income-generating activities to enhance adequate dietary diversification during pregnancy.

**Trial registration:**

Clinicaltrials.gov (PACTR202201731802989, Retrospectively registered on 24 January 2022).

**Supplementary Information:**

The online version contains supplementary material available at 10.1186/s12937-023-00907-z.

## Introduction

Dietary diversity is needed in pregnant women to reduce maternal mortality and morbidity while also laying the framework for fetal growth and perinatal outcome issues [1]. Inadequate dietary diversity during pregnancy, on the other hand, raises the risk of maternal anemia, miscarriage, low birth weight (LBW), preterm birth (PTB), intrauterine growth restriction (IUGR), prenatal and infant mortality, morbidity, and an elevated risk of chronic disease later in life [2, 3]. The incidence of nutritional inadequacy among pregnant women remains a significant problem in sub-Saharan Africa (SSA) [4].

The percentage of pregnant women who eat a diversified diet ranges from 12.8 to 74.5% [5, 6] in Ethiopia. Socio-demographic and economic parameters, food insecurity, a lack of nutrition counseling, and environmental factors were all prevalent predictors of inadequate dietary diversity in low- and middle-income countries (LMICs) [7, 8].

Fruit and vegetable (FV) consumption is recommended as part of a nutrient-dense diet; nevertheless, intakes are frequently lower than recommended levels, including during pregnancy globally [9]. Furthermore, the most common public health issues in Ethiopia are macronutrient and micronutrient deficiencies in pregnant women, and FV is a food group that is often low. Over 80% of Ethiopian women of childbearing age are inadequate in vitamin A, the source of which is primarily FV. The consumption of vitamin-A-rich FV in the overall diet among women in the Oromia region and Addis Ababa was only 3.9% and 2.8%, respectively. Whereas, the consumption of other FV among women in the Oromia region and Addis Ababa was 8.4% and 10.4%, respectively [10].

The key factor in inadequate nutritional intake is pregnant women’s lack of awareness about food needs during pregnancy [11]. The Ethiopian government recommends nutrition counseling throughout pregnancy [12], since improving maternal diet is the most appealing and long-lasting method of improving maternal and child health [13]. Nutrition education is provided to pregnant women by health professionals and health extension workers during antenatal care (ANC) visits and in the community. Despite this, the health-care system’s existing nutrition education is ineffective in changing behavior [14].

Health promotion programs that include behavior change tactics, home visits, and participatory campaigns have been shown to assist women in maintaining their health throughout their reproductive years [15]. Health promotions based on behavioral models and theories have had a positive impact on pregnant women’s dietary behaviors [16]. In this study, the health belief model (HBM) and the theory of planned behavior (TPB) were used to counsel pregnant women [17].

For pregnant women in Ethiopia, intervention using nutrition education models and theories is advocated [14, 18–21], such as education on the consumption of nutrient-rich, locally available foods and food and nutrient supplementation (for example, iron-folic acid [IFA], calcium, and multiple micronutrients), as well as on weight during pregnancy to ensure a healthy weight gain [22]. Therefore, we aimed to assess the effect of a nutrition education intervention on dietary diversity scores among pregnant women in urban settings in Southeast Ethiopia.

## Methods

### Study design, setting, and subjects

A community-based, two-arm, parallel cluster randomized controlled trial study was used among pregnant women attending antenatal care at health facilities in Robe and Goba Towns, Bale Zone, Southeast Ethiopia, from February to December 2021. Robe and Goba Towns were located 430 and 444 km away from Addis Ababa, Ethiopia, respectively. There are 4 hospitals, namely Goba Referral Hospital, Robe, Delomena, and Ginnir; 87 health centers, and more than 300 health posts in the Bale Zone. There were 1,832 and 2,048 pregnant women in the towns of Goba and Robe, respectively. Cluster randomization was used over individual-level randomization to reduce contamination and for pragmatic reasons, as urban health extension workers (UHEWs) operate in clusters [23].

All pregnant women receiving ANC in the towns of Robe and Goba in the Bale Zone were source populations. All first- and early-second-trimester pregnant women who visited ANC clinics in the Robe and Goba Towns, in the Bale Zone, were the study population. First-trimester pregnant women who resided permanently in the study areas were included in the study. Pregnant women who had been diagnosed with HTN, or diabetes mellitus, were excluded from the study.

### Sample size determination and sampling techniques

The sample size was determined using the G-Power software version 3.1, assuming an effect size of 0.25, a 95% confidence level, a precision of 0.05, and a power (1- β) of 80% [24]. The computed sample size was 206. Taking the largest sample size and considering the design effect (DE) of 2 and the 10% attrition rate, the final sample size was 454 (intervention group = 227 and control group = 227).

Two towns, namely, Robe and Goba, were selected. Robe town has three kebeles (the smallest administrative unit) (Baha Biftu, Donsa, and Oda Robe), whereas Goba Town has an Eastern and Western kebeles. The number of pregnant women in each cluster was determined using birth data prepared by UHEWs. Clusters were randomized to the intervention and control groups randomly. Pregnant women in Robe Town received nutrition education, while those in the Goba town control group did not. The sample size was allocated to each cluster by probability proportional to size (PPS). A multistage cluster sampling technique was used to select the pregnant women. The sample size was allocated to each cluster by probability proportional to size (PPS). Pregnant women were selected using a systematic sampling technique. If a woman was absent from her house during the interview, eligible pregnant women in the next house in the serial number were interviewed. The house of the absent pregnant woman was revisited the next day. By asking about the first date of the last menstrual period and confirming pregnancy with a pregnancy test, gestational age was computed.

### Randomization, intervention allocation, and blinding

Pregnant women were assessed for eligibility and allocated randomly to the intervention and control groups using a 1:1 ratio. The allocation sequence was generated using coin tossing. A simple randomization approach, coin-tossing, was used to generate the allocation sequence. The local languages, Afan Oromo and Amharic, were used for communication during nutrition education. The intervention group received nutrition education, counseling cards, and a structured work schedule. The key messages for the lessons were created using the health belief model (HBM) and theory of planned behavior (TPB) theoretical principles [17, 25]. The HBM is an interpersonal health model that is used to foster positive behavior [26]. It explains why some people adopt health-promoting steps while others do not [17, 26]. Prediction of intention and behavior was also conducted using the TPB constructs [17, 25]. TPB was utilised to identify the psychological processes (behavior mediators) that lead to intervention results, both for assessment and to explain dietary behavior changes. The purpose of the study was not to test the theoretical model but to offer direction on the sorts of factors and processes that may be essential in influencing maternal health behaviors and should be addressed in the intervention. The intervention was intended to increase personal and normative nutrition actions [27] towards diverse diet sustenance as a consequence of good nutrition attitudes and behavior control. It was adapted on the recommendation of the Ministry of Health (MOH), Ethiopia [28]. Baseline and end-line assessments were made for two groups. After baseline data collection, pregnant women in the intervention received nutrition education every trimester consistently for six months (six sessions) after they were recruited at their homes in each cluster. Recruitment was done when animal-source foods (ASF) were allowed. Nutrition education was delivered for 30 to 45 min per session. The nutrition education and supervision processes took place with six nurses holding Bachelor of Science (BSc) degrees and two Master of Public Health (MPH) specialists, respectively.

The core content of the session was: increasing knowledge about essential nutrition actions (ENA), iron-rich food sources to improve Hgb, iron-folate acid supplementations (IFAS), iodized salt, meal frequency, and portion size with increasing gestational age; dietary diversifications: food groups; iron absorption enhancers and inhibitors; reducing heavy workloads, taking days off, exercising, increasing utilization of health services, and interrupting the intergenerational life cycle of malnutrition; increase pregnant women’s perceptions of under nutrition and factors leading to it, poor eating practices causing inadequate dietary intake and disease; gestational weight gain, birth weight, food security, a food-based approach; dietary modification; increasing knowledge about FV consumption through diversification, enrichment, and standardization; identifying barriers and ways to overcome them; decreasing the perceived barriers to preparing a FV; encouraging participants to identify ways to overcome the barriers; specific food taboos (meat and eggs); increasing knowledge and attitudes about pregnant women’s ability to modify feeding practices; improving participants’ perceptions of control and intention; and improving participants’ skills in washing hands.

Presentation, discussion, demonstration, and picture-based activities were used to deliver counselling. Compliance was examined using observation, self-report, and attendance. Second-degree public health specialists led the counselling session. Trainers monitored compliance with regards to observation and attendance. They identified critical counseling skills, key messages, feasible activities, and the GALIDRAA (greet, ask, listen, identify, discuss, recommend, agree, set follow-up appointment) processes. Intervention and control groups received nutrition education routinely from nurses. However, due to the unique characteristics of the cluster RCT and the nature of the intervention under research, no concealment was used in the study. This study was not blinded, as the two towns were far apart from each other (more than 10 km). Counseling was not administered by the same nurses to avoid information contamination between intervention and control.

Pregnant women were aware of the intervention; however, pregnant women in both arms were blinded to the research hypotheses. Once the women were enrolled, reasonable efforts were made to promote their retention and complete follow-up by offering soaps to them to minimize missing data. Interest in the study was also maintained through periodic discussions concerning compliance with the intervention during regular meetings as well as home visits by trainers. Furthermore, home visits were scheduled to reduce the pregnant women’s burden associated with follow-up visits. Monitoring and evaluation of the activities were done by the lead author and supervisors.

The control groups were not given a specific schedule. Nevertheless, they received standard healthcare. The control group received a brief intervention at the end of the study to ensure justice and obtain a high degree of post-recruitment satisfaction. In Ethiopia, the MOH’s routine health extension programme packages have 16 components consisting of: family health (family planning, maternal and child health, nutrition, and vaccination services), disease prevention and control (HIV/AIDS and STIs, tuberculosis, malaria, and first aid cares), hygiene and sanitation (promotion of sanitary latrines, waste disposal management, water supply, food hygiene and safety, control of insects and rodents, personal hygiene, and healthy home environment services), and health education [29].

### Data collection

An interviewer-administered, structured questionnaire was used to collect the data. The data collection tools were adapted from the Ethiopian Demographic and Health Survey (EDHS) and existing literature [3, 18, 30]. Data on socio-demographic and economic factors, substance abuse (tea or coffee, alcohol, smoking), and reproductive history were collected before the intervention. Nutritional, intimate partner violence (IPV), physical exercise, health care delivery systems, knowledge and practice of HBM, and TPB tools were collected before and after intervention. End-line data were collected at the end of 36 weeks of gestation.

The principal component analysis (PCA) was used to generate a wealth index. Twenty-one variables entered into the PCA included the availability of a water source, a latrine, a bank account, different types of living houses, livestock, agricultural ownership, and household asset items [31, 32]. The responses of all non-dummy variables were dichotomized as “improved” (= 1) and “unimproved” (= 0) based on the WHO/UNICEF definition. Household income was classified into tertiles: “high,” “medium,” and “poor” [33]. Finally, the highest tertile was designated as having a high income, while the two lowest tertiles were designated as having a low income.

Assumptions of PCA include the correlation matrix for the variables containing two or more correlations ≥ 0.30; variables with measures of sampling adequacy less than 0.50 that must be removed (looking anti-image); the overall measures of sampling adequacy, Kaiser-Meyer-Olkin (KMO) ≥ 0.5 [34], the Bartlett test of sphericity (*p*-value < 0.05); communality > 0.5; and not having the complex structure of correlation ≥ 0.40. Components that collectively explain more than 60% of the variance in the set of variables and have eigenvalues ≥ 1 [35, 36] were used to identify variables to be included in further analyses. Then, the economic status of study subjects was categorized into tertiles as rich, medium, and poor. The highest tertile was used to designate a more wealth lifestyle, while the two lowest tertiles were used to represent a less wealthy lifestyle.

The status of household food security was evaluated using 27 previously validated questions. Food secure, mildly, moderately, and severely food insecure families were defined as having fewer than the first two, two to ten, eleven to seventeen, and more than seventeen food insecurity indicators, respectively [37].

Each HBM was assessed using the sums of a 5-point Likert scale (1 = strongly disagree to 5 = strongly agree) to form a composite question: perceived susceptibility (3 items), perceived severity, and perceived benefits (4 items each), perceived barriers (5 items), cues to action, and self-efficacy (4 items each) [25]. TPB constructs: attitude and subjective norms (3 items each), perceived behavioral control (2 items), and behavioral intention (2 items) (7 items) [38]. The factor scores were summed and categorized into tertiles, and the highest tertile was labelled as having a “yes” for perceived susceptibility, severity, benefit, barriers, cues to action, self-efficacy, positive attitude, perceived behavioral control, and behavioral intention; otherwise, the two lower tertiles were labelled as “no.” A ten-item knowledge questions was also used to measure the significance of fruits and vegetables [39]. If a respondent answered correctly, they received a 1; otherwise, they received a 0. After that, the scores were computed and ranked in tercile order. Finally, the highest tertile was labeled with a high level of nutrition knowledge, while the two lower tertiles were labeled with a poor level of nutrition knowledge.

### Data quality control

To ensure the quality of the data, the questionnaire was initially developed in English and then translated into the local languages (*Afan Oromo and Amharic)* and back-translated into English by independent language experts to ensure its consistency. The questionnaire was also pretested on 5% of the total estimated sample size, which had similar characteristics to the study subjects in a different adjacent setting.

An epidemiologist and a biostatistician reviewed the completed questionnaire to ensure its validity. Finally, we modified the questionnaire based on the findings of the pre-test and specialty reviews (setting and wording questions, sequencing, formatting, and scaling responses). The training was given to eight nurses with BSc degrees as data collectors and four MPH specialists as supervisors on the objectives of the study, data collection instruments, and ethical issues to minimize interviewers’ bias. Six counselors were trained by the lead author. A lead author met with counselors every two weeks to discuss ways of improving the counseling session process.

Supervisors closely followed the data collectors daily for the successful completion of the questionnaire and took timely action in case of any deviations. The study participants were interviewed at their homes to improve the response rate. The internal consistency of the cognitive, affective, and psychomotor domains of the tools were assessed using Cronbach’s alpha (Table S1).

### Outcome assessment

The dietary diversity score (DDS) was the primary outcome variable. A 24-hour qualitative dietary recall method was used to calculate the DDS. The DDS for women is a nine-food group to indicate a qualitative indicator of micronutrient sufficiency in a diet [40]. The score was calculated using nine food classes in order to indicate the diet’s micronutrient sufficiency. Participants were asked to recall everything they had eaten and drunk in the previous 24 h, both inside and outside their homes.

Participants were also asked to recall any snacks they had had in between big meals. Consumption of a food item during the reference period was assigned a score of “1,” whereas non-consumption was assigned a score of “0.” The foods were then classified into nine groups: (1) starchy staples; (2) dark green leafy vegetables; (3) vitamin-A-rich fruits and vegetables; (4) other fruits and vegetables; (5) beans, nuts, and seeds; (6) meat and fish; (7) fats and oils; (8) milk and milk products; and (9) eggs. The DDS was calculated by adding the food groups consumed during the reference period and ranking them into tertiles, with the highest tertile indicating a high DDS (= 1) and the two lower tertiles combined representing a low DDS (= 0) [41].

### Data processing and analysis

The data were checked for completeness, consistency, and accuracy and entered into, cleaned, and analyzed using SPSS for Windows version 20 and Stata version 14. Descriptive statistics, including frequencies, percentages, means, and standard deviations, were generated for the selected predictors. The baseline characteristics of the intervention and control groups were assessed using the chi-square test. The McNemar test was used to compare DDS within the intervention and control groups. The difference in difference (DID) analyses were used to estimate DDS between the two study arms [42].

Variables with a *p*-value less than 0.25 in a bivariable generalized estimating equations (GEE) binary logistic model were entered into a multivariable GEE binary logistic model to account for within-cluster correlation of measurements. GEE can manage missing data values without the need for explicit imputation by considering all study subjects with at least one follow-up visit and automatically removing the missing values via the GEE analysis model [43].

Although the GEE method is thought to be robust against incorrect working correlation structure (WCS) selection, the best WCS of the DDS was chosen as an unstructured matrix by assessing the working correlation matrix of the observed correlations between subsequent measurements to maintain the goodness of the model fitness and thus obtain a more precise estimation of the intervention effect. Accordingly, the quasi-likelihood under the independence model criterion (QIC) was used to maintain the goodness of model fitness. In our data analysis, there were similar results for all working correlation matrixes. The intervention’s effectiveness was determined using time and treatment interactions. The adjusted odds ratio (AOR), along with a 95% confidence interval (CI), was used to estimate the strength of the association between predictors and the endpoint variable. Initially, randomly assigned pregnant women were examined in the groups to which they were assigned (intention-to-treat analysis principle). The statistical significance of the association was declared at a *p*-value of less than 0.05, and all tests were two-sided.

**Fig. 1:**
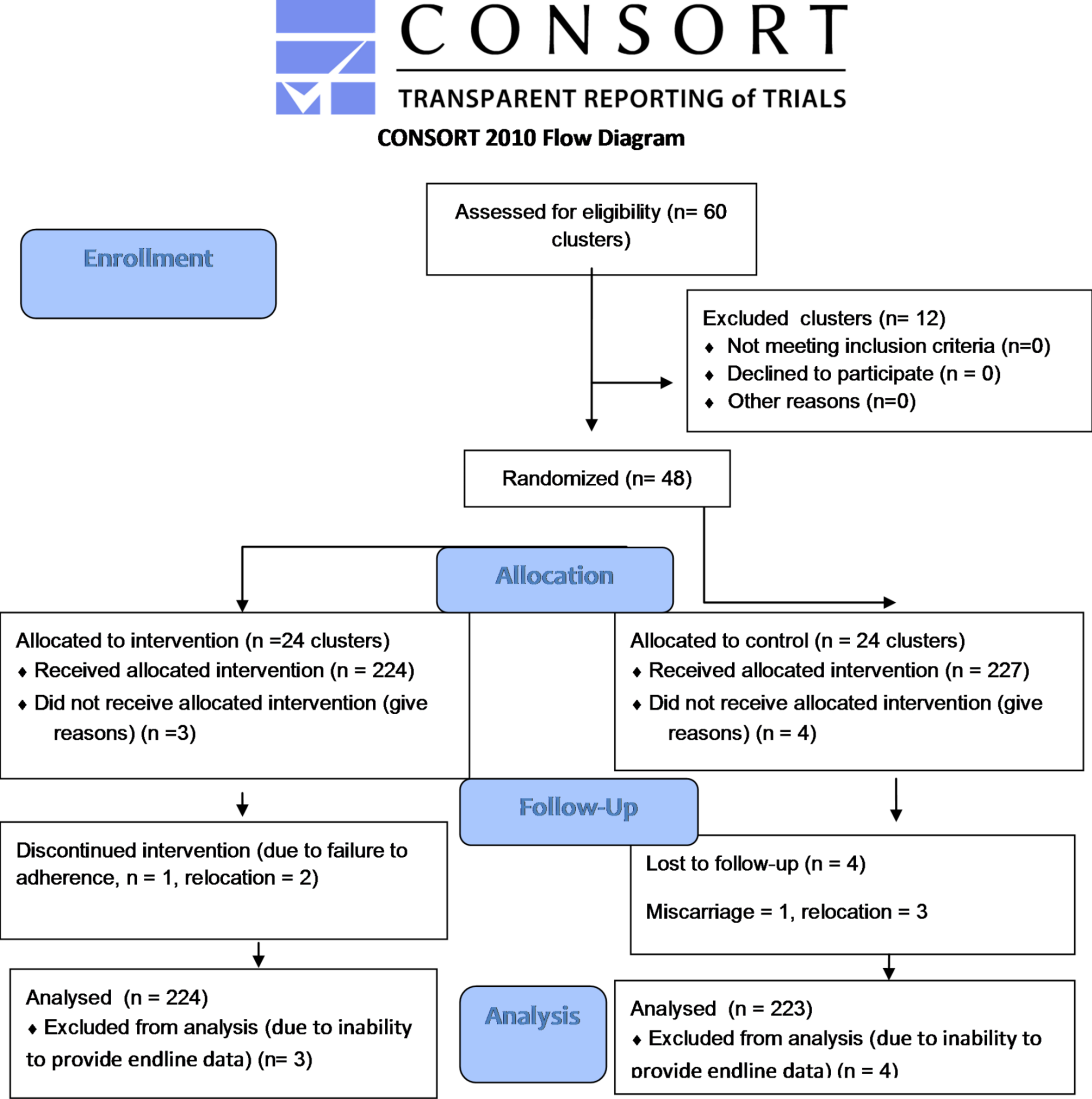
Flow diagram of study subjects

## Results

### Socio-demographic and economic characteristics

A total of 224 (98.67%) and 223 (98.24%) pregnant women were successfully surveyed in the intervention and control groups, respectively (Fig. 1). The mean (± SD) age of the pregnant women was 25.93 (± 5.52) years for the intervention group and 24.24 (± 4.24) years for the control group. There was no statistically significant difference in baseline characteristics between the intervention and control groups (*P*-value > 0.05) except in ethnicity (Table 1).

**Table 1:** Baseline socio-demographic and economic characteristics of pregnant women in Southeast Ethiopia, using the chi square test, 2020/21

Variables	Control group (n = 223) Frequency (%)	Intervention group (n = 224) Frequency (%)	*P*-value
**Age (years)**			
< 20	27 (55.1)	196 (49.25)	0.44
≥ 20	22 (44.90)	202 (50.75)	
**Marital status**			
Married	222 (99.55)	223 (99.55)	0.99
Unmarried	1 (0.45)	1 (0.45)	
**Religion**			
Orthodox	112 (50.22)	101 (45.09)	0.58
Muslim	88 (39.46)	102 (45.54)	
Protestant	21 (9.42)	20 (8.93)	
Others #	2 (0.90)	1 (0.45)	
**Ethnicity**			
Oromo	187 (83.86)	193 (86.16)	**0.03**
Amhara	33 (14.80)	21 (9.38)	
Others ◱	3 (1.35)	10 (4.46)	
**Occupation status**			
Housewife	139 (62.33)	124 (55.36)	0.14
Government employee	46 (20.63)	52 (23.21)	
NGO employee	3 (1.35)	13 (5.80)	
Petty trade	22 (9.87)	22 (9.82)	
Student	9 (4.04)	11 (4.91)	
Daily laborer	4 (1.79)	2 (0.89)	
**Maternal education**			
Literate	209 (93.72)	212 (94.64)	0.68
Illiterate	14 (6.28)	12 (5.36)	
**Spouse education**			
No formal education	31 (13.90)	24 (10.71)	0.20
Primary education	52 (23.32)	39 (17.41)	
Secondary education	66 (29.60)	82 (36.61)	
Tertiary education	74 (33.18)	79 (35.27)	
**Wealth index**			
More wealthy	66 (29.60)	76 (33.93)	0.33
Less wealthy	157 (70.40)	148 (66.07)	
**Family size**			
< 5	197 (88.34)	188 (83.93)	0.18
≥ 5	26 (11.66)	36 (16.07)	
Food security			
Food insecure	89 (39.91)	82 (36.61)	0.47
Food secured	134 (60.09)	142 (63.39)	

# Others: ◱Wakafeta; Others: Gurage, Hadiya; NGO: Non-governmental organization

### Health belief model and the theory of planned behavior scores

There was significant improvement in the score of HBM and TPB constructs except for perceived benefit and cues to actions among the intervention group before and after the intervention (*P*-value < 0.0001). Furthermore, with the exception of perceived severity and cues to action, there was a significant difference in the dimensions of HBM and TPB in the end-line data (Table 2). The HBM and TPB constructs showed that perceived susceptibility, perceived benefits, perceived barriers, self-efficacy, and TPB were all correlated with interventions. However, there was no correlation between dietary diversification and HBM and TPB constructs (Table S2).

**Table 2:** Comparison of the health belief model and the theory of planned behavior constructs between and within the intervention and control groups using an independent t test and a paired t test during pregnancy in Southeast Ethiopia, 2021

HBM and TPB constructs	Study period	HBM and TPB constructs score		*P*
		Control group, Mean ± SD	Intervention group, Mean ± SD	
Perceived susceptibility	Baseline Endline *P*	5.27 ± 2.2 5.46 ± 1.95 0.04	5.33 ± 2.48 7.43 ± 1.74 < 0.0001	0.80 < 0.0001
Perceived severity	Baseline Endline *P*	8.05 ± 2.70) 8.57 ± 2.49) < 0.0001	7.34 ± 2.72 8.30 ± 2.36 < 0.0001	0.006 0.25
Perceived Benefit	Baseline Endline *P*	11.04 ± 1.66 10.86 ± 1.79 0.10	11.46 ± 1.42 11.57 ± 1.22 0.07	0.004 < 0.0001
Perceived barriers	Baseline Endline *P*	8.55 ± 2.71 9.25 ± 2.88 < 0.0001	8.20 ± 2.43 7.88 ± 2.54 0.02	0.39 < 0.0001
Cues to action	Baseline Endline *P*	11.0 ± 2.61 11.61 ± 1.44 < 0.0001	11.65 ± 1.15 11.55 ± 1.19 0.25	< 0.0001 0.57
Self-efficacy	Baseline Endline *P*	9.22 ± 2.07 9.55 ± 11.81 < 0.0001	9.93 ± 2.28 10.58 ± 1.69 < 0.0001	0.001 < 0.0001
Attitude	Baseline Endline *P*	13.32 ± 1.66 13.61 ± 1.69 0.002	12.32 ± 3.84 14.23 ± 1.49 < 0.0001	< 0.0001 < 0.0001
Subjective norm	Baseline Endline *P*	13.46 ± 1.55 13.80 ± 1.39 < 0.0001	12.89 ± 3.71 14.29 ± 1.51 < 0.0001	0.04 < 0.0001
Perceived behavioral control	Baseline Endline *P*	7.48 ± 2.25 8.17 ± 1.92 < 0.0001	7.92 ± 2.44 9.37 ± 1.40 < 0.0001	0.04 < 0.0001
Behavioral intention	Baseline Endline *P*	31.62 ± 3.52 32.91 ± 2.39 < 0.0001	30.30 ± 8.07 33.64 ± 2.71 < 0.0001	0.02 0.003

HBM: Health belief model; TPB: Theory of planned behavior; P: *P*-value; SD: Standard deviation

### Food taboos during pregnancy

The proportion of pregnant women who had religious food restrictions was 12.9% (95% CI: 8.3, 16.4%) and 13.9% (95% CI: 8.8, 18.90%) in the intervention and control groups, respectively. Butter, 14 (6.3%), bananas, 6 (2.7%), and milk, 5 (2.2%), were considered dietary taboos, followed by beef, 3 (1.3%), and eggs, 1 (0.4%), in the intervention group, whereas milk, 10 (4.5%), butter, 7 (3.1%), banana, 5 (2.2%), eggs, 5 (2.2%), and beef, 4 (1.8%), in the control group. The two groups were comparable in terms of maternal education and food taboos at baseline.

The proportion of pregnant women who had religious food restrictions decreased to 6.7% (95% CI: 3.3, 9.7%) in the intervention group but remained high at 12.6% (95% CI: 8.0, 17.10%) in the control groups at end-line (*P* = 0.03). Butter, 7 (3.1%), milk, 4 (1.8%), and bananas, 3 (1.3%), were considered dietary taboos, followed by eggs, 1 (0.4%), in the intervention group, whereas milk, 9 (4.0%), butter, 6 (2.7%), eggs, 5 (2.2%), banana, 4 (1.8%), and beef, 4 (1.8%), in the control group.

### Dietary diversity score

Before the intervention, the proportion of adequate dietary diversity (DD) in the two arms was (intervention, 37.50% versus control, 31.84%). At baseline, there was no statistically significant difference in DDS between the two groups (*P* = 0.21). After the intervention, the proportion of adequate DD was 14.15% higher in the intervention arm compared to the control group (45.09% versus 30.94%, *P* = 0.002) (Table 3). The overall difference in adequate DD between the groups was 8.5%. The difference in differences (DID) indicated that there was a statistically significant difference between the intervention and control groups (Table 4).

**Table 3:** Comparison of the food groups between the intervention and control groups during pregnancy using the chi square test in Southeast Ethiopia, 2021

Food groups	Study period	Category	Control group, Frequency (%)	Intervention group, Frequency (%)	*P*-value
Cereals	Baseline End line	Yes	223 (100)	224 (100)	-
		Yes	223 (100)	224 (100)	
Vitamin A rich FV	Baseline End line	Yes	139 (62.33)	129 (57.59)	0.30
		No	84 (37.67)	95 (42.41)	
		Yes	146 (65.47)	165 (73.66)	0.06
		No	77 (34.53)	59 (26.34)	
Other fruit	Baseline End line	Yes	113 (50.67)	91 (40.63)	0.03
		No	110 (49.33)	133 (59.38)	
		Yes	128 (57.40)	132 (58.93)	0.74
		No	95 (42.60)	92 (41.07)	
Other vegetable	Baseline End line	Yes	94 (42.15)	107 (47.77)	0.23
		No	129 (57.85)	117 (52.23)	
		Yes	98 (43.95)	128 (57.14)	0.005
		No	125 (56.05)	96 (42.86)	
Legumes and Nut	Baseline End line	Yes	110 (49.33)	224 (53.57)	0.37
		No	113 (50.67)	104 (46.43)	
		Yes	116 (52.02)	134 (59.82)	0.09
		No	107 (47.98)	90 (40.18)	
Meat, poultry, & fish	Baseline End line	Yes	71 (31.84)	77 (34.38)	0.57
		No	152 (68.16)	147 (65.63)	
		Yes	86 (38.57)	110 (49.11)	0.03
		No	137 (61.43)	114 (50.89)	
Fats and oils	Baseline End line	Yes	207 (92.83)	216 (96.43)	0.09
		No	16 (7.17)	8 (3.57)	
		Yes	213 (95.52)	223 (99.55)	0.006
		No	10 (4.48)	1 (0.43)	
Dairy products	Baseline End line	Yes	83 (37.22)	98 (43.75)	0.16
		No	140 (62.78)	126 (56.25)	
		Yes	85 (38.12)	109 (48.66)	0.03
		No	138 (61.88)	115 (51.34)	
Eggs	Baseline End line	Yes	57 (25.56)	53 (23.66)	0.64
		No	166 (74.44)	171 (76.34)	
		Yes	64 (28.70)	77 (34.38)	0.20
		No	159 (71.30)	147 (65.63)	
DDS	Baseline End line	High	71 (31.84)	84 (37.50)	0.21
		Low	152 (68.16)	140 (62.50)	
		High	69 (30.94)	101 (45.09)	0.002
		Low	154 (69.06)	123 (54.91)	

DDS: Dietary diversity score; FV: Fruit and vegetable

**Table 4:** Differences between baseline and end-line measurements of DDS and difference of the differences between the intervention and control groups using a McNemar test

Group	Baseline	End line	Difference (EL-BL)	Difference of difference	*P*-value
Intervention	0.375	0.451	0.076	0.085 ± 0.05	< 0.0001
Control	0.318	0.309	-0.009		

BL: Baseline; EL: End line

### Prediction of adequate dietary diversity among pregnant women

The multivariable generalized estimating equations (GEE) logistic regression model revealed that having received intervention [(AOR = 1.89, 95% CI: (1.27, 2.79)], being literate [(AOR = 3.41, 95% CI: (1.13, 10.23)], and having a high wealth quintile [(AOR = 1.60, 95% CI: (1.09, 2.35)] significantly improved adequate dietary diversity (Table 5).

**Table 5:** Multivariable generalized estimating equations logistic regression model predicting adequate dietary diversity among pregnant women

	*P*	AOR	95% CI	
			Lower	Upper
Intercept	< 0.0001	0.042	0.010	0.188
**Maternal age**				
≥ 20	0.910	1.040	0.490	2.220
< 20		Ref		
**Maternal education**				
Literate	0.030	**3.410**	1.130	10.230
Illiterate		Ref		
**Wealth index**				
More wealthy	0.020	**1.600**	1.090	2.350
Less wealthy		Ref		
**Nutrition counsel**				
Yes	0.310	1.240	0.820	1.860
No		Ref		
**Have adequate money to buy food** Yes	0.997	1.001	0.620	1.620
No		Ref		
**End line knowledge on FV**				
high knowledge	0.810	1.050	0.710	1.530
low knowledge		Ref		
**End line food security**				
Food secure	0.595	1.100	0.760	1.610
Food insecure		Ref		
**End-line organ meat**				
Yes	0.400	0.850	0.580	1.230
No		Ref		
**Group type**				
Intervention	0.890	0.970	0.620	1.500
Control		Ref		
Time [End line]	0.010	1.410	1.080	1.850
Time [baseline]		Ref		
Time [end line] * Intervention	0.001	**1.890**	1.270	2.790

Model was adjusted for maternal age, maternal education, wealth index, baseline nutrition counsel, adequate money to buy food, End line knowledge on FV, end line food security status, end line organ meat, time, group type, time * group type,

P: *P*-value; AOR: Adjusted odds ratio; CI: Confidence interval; SE: standard error; Ref: Reference category, FV: Fruit and vegetable; Time: Follow-up rounds; Maximum SE = 0.55

## Discussion

We found that the nutrition education intervention achieved a significant improvement in the consumption of foods as shown by an increase in dietary diversity score in the intervention arm compared to controls. The results showed that having received the intervention, being literate, and having a high wealth quintile significantly improved adequacy of dietary diversity.

After controlling the potential confounders, the pregnant women in the intervention group were 1.89 times more likely to have adequate dietary diversity compared to their counterparts. As health professionals frequently contacted pregnant women in their homes in this current research, this might explain why there was an enhanced intake of dietary diversity. The findings of this study adds to a growing body of evidence in Ethiopia [14, 21, 39, 44, 45], as well as in other medium-and low-income countries such as Burkina Faso and Malawi [46–48], Iran, and Pakistan [49, 50]. The possible explanation could be that women in these countries have low levels of awareness of the importance of dietary diversity and that education that includes the HBM and TPB dimensions will improve this knowledge. This is consistent with the finding of a study conducted in West Gojjam, Ethiopia, which found that the HBM and TPB dimensions had a significant positive association with the DDS during pregnancy [21]. This builds the case that applying health behavior models and theories to behavior change communication improves dietary behaviors.

Increased behavioral changes towards DDS may indicate the intervention’s effectiveness in creating favorable beliefs and expectations about optimal nutrition during pregnancy. Moreover, education and counseling can assist women in realizing the value of a varied diet throughout pregnancy and encourage continuous efforts to maintain a broad diet [51]. In addition, health promotion-based educational interventions can be beneficial in raising awareness, improving understanding of risks, removing obstacles to healthy behavior, and, ultimately, improving women’s health and nutritional performance throughout pregnancy [52].

Literate pregnant women were 3.41 times more likely to eat a diet with adequate dietary diversity compared to those who had no formal education. This study’s findings agreed with the reports of studies conducted in other parts of Ethiopia [53], Kenya [54], and Bangladesh [55]. This could be due to the way food taboos influence consumption. On the other hand, educated women might have more employment opportunities and wealth, which may increase their consumption of a variety of meals. Pregnant women who were in the high-wealth quintile were 1.60 times more likely to consume a diet with adequate diversity. These findings are in accordance with evidence from other studies conducted in Ethiopia [18, 56] and Kenya [54]. One possible explanation is that pregnant women from the wealthier quintile had access to healthier foods and more diversity in their diets.

The proportion of pregnant women who had religious food restrictions was 12.9% and 13.9% in the intervention and control groups, respectively. Butter, bananas, and milk were considered food taboos, followed by beef and eggs, in the intervention group, whereas milk, butter, bananas, eggs, and beef were considered food taboos in the control group at baseline. This study’s finding was lower than other studies conducted in Ethiopia [57–63], in which many pregnant women reported having food taboos. This finding agreed with studies conducted in Kenya, Kwazulu-Natal, South Africa, and India [64–66].

The proportion of women who had food taboos was 6.7% in the intervention group. Butter, milk, and bananas were considered food taboos, followed by eggs, in the intervention group, whereas milk, butter, eggs, bananas, and beef were considered food taboos in the control group at the end line. This decrease in food taboos may be due to the positive effect of education.

The strength of this study is its community-based, cluster-randomised, controlled trial design, in which promoting fruit and vegetable consumption and other health messages were integrated with HBM and TPB, which are applicable to relevant and standard ANC. For generalizability, cluster randomized controlled trials should have both internal and external validity [67]. Both the sample size determination and the data analysis took the cluster nature of the study into account. Adoption, or the degree to which the setting is typical of the larger population, and program implementation evaluation could also be used to assess external validity [67].

However, the findings of this study could have been influenced by recall bias and social desirability bias. Nonetheless, efforts were made to probe pregnant women on multiple passes for 24 h in order to improve recall. Self-reporting, on the other hand, is often employed in nutrition assessments and has been demonstrated to have greater behavior prediction power than objective assessments [68]. Pregnant women in the control group had not had the same number of visits as those in the intervention group. While the intervention was trimester-based, promoting dietary diversity consumption during pregnancy should be initiated during the preconception period to prevent adverse pregnancy outcomes (APO). Furthermore, this study’s findings could not be generalized to pregnant women living in rural settings in Southeast Ethiopia, as it was conducted among pregnant women residing in urban areas in Southeast Ethiopia.

## Conclusion

The findings showed that having received intervention, being literate, and being in the high wealth quintile significantly improved adequate dietary diversity. Efforts should be made to increase nutrition education using the health belief model (HBM) and the theory of planned behavior (TPB). As well, other theories such as stages of change (the trans-theoretical model), might also be of value.

Moreover, there is a need to improve literacy and economic empowerment through income-generating activities to enhance adequate dietary diversification during pregnancy. Further studies are needed to assess the impact of nutrition education interventions delivered during pregnancy.

### Electronic supplementary material

Below is the link to the electronic supplementary material.

**Supplementary Material 1: Table S1:** Internal consistency of knowledge, HBM, TPB, and practice during pregnancy, Southeast Ethiopia. **Table S2:** Comparison of Correlation of the HBM and the TPB dimensions with knowledge, Hgb, DDS, and MUAC among pregnant women in Southeast Ethiopia.

**Supplementary Material 2: Table 3:** Table S1 CONSORT checklist

## Data Availability

All relevant data for this study are available upon reasonable request from the corresponding author.
