# Supplementary material for: Effect of nutrition education integrating the health belief model and theory of planned behavior on dietary diversity of pregnant women in Southeast Ethiopia: a cluster randomized controlled trial
Source: Nutr J. 2024 Jan 3;23:3. doi: 10.1186/s12937-023-00907-z (PMC10763129; doi:10.1186/s12937-023-00907-z)
Supplement: Supplementary file 1 — Supplementary Material 1: Table S1: Internal consistency of knowledge, HBM, TPB, and practice during pregnancy, Southeast Ethiopia. Table S2: Comparison of Correlation of the HBM and the TPB dimensions with knowledge, Hgb, DDS, and MUAC among pregnant women in Southeast Ethiopia. [file 12937_2023_907_MOESM1_ESM.docx]

**Supportive information**

Table S1: Internal consistency of knowledge, HBM, TPB, and practice during pregnancy, Southeast Ethiopia

| Knowledge, HBM, TPB, & practice |  | Control group | Intervention group |  |
| --- | --- | --- | --- | --- |
|  |  | Cronbach’s Alpha | | No of items |
| Knowledge | Baseline | 0.77 | 0.72 | 10 |
|  | Endline | 0.84 | 0.73 | 10 |
| Perceived susceptibility | Baseline | 0.94 | 0.91 | 3 |
|  | Endline | 0.71 | 0.71 | 3 |
| Perceived seriousness | Baseline | 0.86 | 0.81 | 4 |
|  | Endline | 0.79 | 0.71 | 4 |
| Perceived benefit | Baseline | 0.73 | 0.75 | 4 |
|  | Endline | 0.72 | 0.73 | 4 |
| Perceived barrier | Baseline | 0.87 | 0.75 | 5 |
|  | Endline | 0.71 | 0.72 | 5 |
| Cues to action | Baseline | 0.71 | 0.71 | 4 |
|  | Endline | 0.90 | 0.72 | 4 |
| Self-efficacy | Baseline | 0.89 | 0.88 | 4 |
|  | Endline | 0.72 | 71 | 4 |
| Attitude | Baseline | 0.90 | 0.92 | 3 |
|  | Endline | 0.73 | 0.72 | 3 |
| Subjective norm | Baseline | 0.94 | 0.96 | 3 |
|  | Endline | 0.77 | 0.73 | 3 |
| Perceived behavioral control | Baseline | 0.83 | 0.84 | 2 |
|  | Endline | 0.71 | 0.75 | 2 |
| Behavioral intention | Baseline | 0.95 | 0.96 | 7 |
|  | Endline | 0.72 | 0.74 | 7 |
| Practice | Baseline | 0.71 | 0.73 | 13 |
|  | Endline | 0.71 | 0.73 | 13 |

HBM: Health belief model; TPB: Theory of planned behavior

Table S2: Comparison of Correlation of the HBM and the TPB dimensions with knowledge, Hgb, DDS, and MUAC among pregnant women in Southeast Ethiopia

|  | Intervention | Perceived susceptibility | Perceived severity | Perceived benefit | Perceived barrier | Cues to actions | Self-efficacy | Attitude | Subjective norm | PBC | Behavior intention | Knowledge | Hgb | MUAC | DDS |
| --- | --- | --- | --- | --- | --- | --- | --- | --- | --- | --- | --- | --- | --- | --- | --- |
| Intervention | 1 | .472^**^ | -.054 | .226^**^ | -.243^**^ | -.026 | .282^**^ | .189^**^ | .167^**^ | .336^**^ | .141^**^ | -.005 | .139^**^ | .327^**^ | .123^**^ |
|  |  | .000 | .256 | .000 | .000 | .578 | .000 | .000 | .000 | .000 | .003 | .908 | .003 | .000 | .009 |
| Perceived susceptibility | .472^**^ | 1 | .218^**^ | .023 | .055 | .084 | .047 | .030 | .111^*^ | .172^**^ | .133^**^ | .118^*^ | .033 | .246^**^ | .076 |
|  | .000 |  | .000 | .629 | .248 | .077 | .325 | .526 | .019 | .000 | .005 | .012 | .490 | .000 | .111 |
| Perceived severity | -.054 | .218^**^ | 1 | .083 | .233^**^ | .021 | .027 | -.078 | -.069 | .102^*^ | -.098^*^ | -.066 | -.113^*^ | .085 | .005 |
|  | .256 | .000 |  | .079 | .000 | .662 | .564 | .099 | .143 | .031 | .038 | .165 | .017 | .074 | .914 |
| Perceived benefit | .226^**^ | .023 | .083 | 1 | -.086 | .086 | .216^**^ | .035 | .020 | .052 | .048 | -.142^**^ | .055 | .166^**^ | .014 |
|  | .000 | .629 | .079 |  | .069 | .070 | .000 | .464 | .679 | .273 | .312 | .003 | .246 | .000 | .774 |
| Perceived barriers | -.243^**^ | .055 | .233^**^ | -.086 | 1 | .083 | -.292^**^ | -.034 | .052 | .007 | .062 | .203^**^ | -.049 | -.070 | -.062 |
|  | .000 | .248 | .000 | .069 |  | .081 | .000 | .473 | .268 | .888 | .194 | .000 | .305 | .139 | .190 |
| Cues to actions | -.026 | .084 | .021 | .086 | .083 | 1 | -.016 | -.011 | .015 | -.014 | .093 | .028 | -.012 | .023 | .033 |
|  | .578 | .077 | .662 | .070 | .081 |  | .744 | .816 | .749 | .772 | .051 | .552 | .803 | .625 | .484 |
| Self-efficacy | .282^**^ | .047 | .027 | .216^**^ | -.292^**^ | -.016 | 1 | -.017 | .003 | .027 | -.038 | -.183^**^ | -.019 | .149^**^ | .068 |
|  | .000 | .325 | .564 | .000 | .000 | .744 |  | .720 | .952 | .569 | .418 | .000 | .682 | .002 | .154 |
| Attitude | .189^**^ | .030 | -.078 | .035 | -.034 | -.011 | -.017 | 1 | .345^**^ | .166^**^ | .237^**^ | .096^*^ | .063 | .072 | .021 |
|  | .000 | .526 | .099 | .464 | .473 | .816 | .720 |  | .000 | .000 | .000 | .043 | .184 | .130 | .653 |
| Subject norm | .167^**^ | .111^*^ | -.069 | .020 | .052 | .015 | .003 | .345^**^ | 1 | .115^*^ | .305^**^ | .099^*^ | .089 | -.051 | -.063 |
|  | .000 | .019 | .143 | .679 | .268 | .749 | .952 | .000 |  | .015 | .000 | .036 | .061 | .279 | .185 |
| PBC | .336^**^ | .172^**^ | .102^*^ | .052 | .007 | -.014 | .027 | .166^**^ | .115^*^ | 1 | .219^**^ | -.023 | .001 | .197^**^ | -.007 |
|  | .000 | .000 | .031 | .273 | .888 | .772 | .569 | .000 | .015 |  | .000 | .625 | .976 | .000 | .887 |
| Behavior intention | .141^**^ | .133^**^ | -.098^*^ | .048 | .062 | .093 | -.038 | .237^**^ | .305^**^ | .219^**^ | 1 | .067 | .042 | .070 | -.080 |
|  | .003 | .005 | .038 | .312 | .194 | .051 | .418 | .000 | .000 | .000 |  | .155 | .375 | .137 | .093 |
| Knowledge | -.005 | .118^*^ | -.066 | -.142^**^ | .203^**^ | .028 | -.183^**^ | .096^*^ | .099^*^ | -.023 | .067 | 1 | .033 | -.008 | .011 |
|  | .908 | .012 | .165 | .003 | .000 | .552 | .000 | .043 | .036 | .625 | .155 |  | .489 | .871 | .825 |
| Hgb | .139^**^ | .033 | -.113^*^ | .055 | -.049 | -.012 | -.019 | .063 | .089 | .001 | .042 | .033 | 1 | .037 | .057 |
|  | .003 | .490 | .017 | .246 | .305 | .803 | .682 | .184 | .061 | .976 | .375 | .489 |  | .436 | .232 |
| MUAC | .327^**^ | .246^**^ | .085 | .166^**^ | -.070 | .023 | .149^**^ | .072 | -.051 | .197^**^ | .070 | -.008 | .037 | 1 | -.048 |
|  | .000 | .000 | .074 | .000 | .139 | .625 | .002 | .130 | .279 | .000 | .137 | .871 | .436 |  | .307 |
| DDS | .123^**^ | .076 | .005 | .014 | -.062 | .033 | .068 | .021 | -.063 | -.007 | -.080 | .011 | .057 | -.048 | 1 |
|  | .009 | .111 | .914 | .774 | .190 | .484 | .154 | .653 | .185 | .887 | .093 | .825 | .232 | .307 |  |
| **. Correlation is significant at the 0.01 level (2-tailed). | | | | | | | | | | | | | | | |
| *. Correlation is significant at the 0.05 level (2-tailed). | | | | | | | | | | | | | | | |

PBC: Perceived behavioral control; Hgb: Hemoglobin; MUAC: Mid-upper arm circumference; DDS: Dietary diversity score
